# Socioeconomic inequalities in child and adolescent mental health in Australia: the role of parenting style and parents’ relationships

**DOI:** 10.1186/s13034-024-00719-x

**Published:** 2024-02-21

**Authors:** Nirmal Gautam, Mohammad Mafizur Rahman, Rubayyat Hashmi, Apiradee Lim, Rasheda Khanam

**Affiliations:** 1 School of Business, University of Southern Queensland, Toowoomba, Queensland 4350 Australia; 2 The Centre for Health Research, The University of Southern Queensland, Toowoomba, Queensland 4350 Australia; 3 The Australian Centre for Housing Research, The University of Adelaide, Adelaide, SA 5005 Australia; 4 NGRN, The ALIVE National Centre for Mental Health Research Translation, The University of Melbourne, Parkville, VIC 3052 Australia; 5 Department of Mathematics and Computer Science, Faculty of Science and Technology, Prince of Songkla University, Pattani, 94000 Thailand

**Keywords:** Socioeconomic inequality, Mental health, Parental style, Couple relationships

## Abstract

**Background:**

Socioeconomic inequalities in health and their determinants have been studied extensively over the past few decades. However, the role of parenting style and parents’ couple relationships in explaining mental health inequalities is limited. Therefore, this study aims to investigate the distributional impact of parenting style (angry parenting, consistent parenting, and inductive parenting) and parents’ couple relationships (e.g., argumentative, happy relationships) on socioeconomic inequalities and by extension on mental health status of Australian children and adolescents.

**Methods:**

This study utilized data from the Longitudinal Study of Australian Children (Waves 1–7), specifically focusing on intact biological parent families, while excluding single-parent and blended-family households. We applied the decomposition index and the Blinder Oaxaca method to investigate the extent of the contribution and temporal impact of parenting style and parents’ couple relationships on the mental health status of Australian children and adolescents.

**Results:**

This study revealed that poor parenting style is the single most important factor that leads to developing mental health difficulties in children and adolescents, especially from low socioeconomic status, and it contributes almost 52% to socioeconomic inequalities in mental health status. Conversely, household income, maternal education, employment status, and parents’ couple relationships contributed 28.04%, 10.67%, 9.28%, and 3.34%, respectively, to mental health inequalities in children and adolescents.

**Conclusion:**

Overall, this study underscores the importance of parenting style and parents’ couple relationships as significant predictors of mental health outcomes in children and adolescents. These results highlight the need for targeted interventions to support families from low socioeconomic backgrounds to address the significant mental health inequalities observed in the study population.

**Supplementary Information:**

The online version contains supplementary material available at 10.1186/s13034-024-00719-x.

## Background

Childhood and adolescence are critical developmental stages that provide the foundation for future health and wellbeing. During these formative years, individuals undergo significant brain development and acquire pivotal social, emotional, and behavioral competencies [1, 2]. However, the trajectory of this development is markedly influenced by socioeconomic status (SES), a multi-dimensional construct encompassing aspects of income, education, and occupation [3, 4]. Disparities in SES are known to create significant variations in the developmental outcomes of children and adolescents, often placing those from lower SES backgrounds at a disadvantage owing to restricted access to resources and opportunities [5, 6].

Research has consistently shown that lower SES is associated with poorer cognitive, social, and emotional development in children [6–8]. This developmental gap arises from a combination of factors, including limited material resources, increased familial stress, and reduced quality of parental care [4, 9]. As such, children and adolescents from low-SES backgrounds are at an elevated risk for negative health outcomes, both in the short and long term [9, 10].

In understanding the complexities of socioeconomic health inequalities, it is crucial to explore the roles of parenting style and parental relationships. Parenting style, a concept introduced by Baumrind [12], refers to the strategies and attitudes parents adopt while raising their children, which in turn shape the emotional and developmental milieu of the family [11]. Parenting styles are broadly categorized as authoritative, authoritarian, and permissive. The authoritative style is characterized by high levels of warmth and consistency. The permissive style involves high levels of warmth and low consistency. On the other hand, the authoritarian style is characterized by low levels of warmth but high levels of consistency, each with distinct impacts on child development [12, 13]. The quality of parental relationships, often characterized by aspects of communication, conflict resolution, and emotional support, further influences the family dynamics and, by extension, the mental health of children and adolescents [14, 15].

Empirical studies have provided insight into these relationships. For example, Pierron et al. [16] conducted a synthesis of systematic reviews revealing the critical role of supportive parenting in mitigating the adverse effects of socioeconomic disparities on children's health. Other studies echoed these findings [16]. For instance, Conger et al. [17] found that economic strains in low-SES families adversely affect parenting styles, leading to increased stress and negative parent–child interactions. In turn, this can contribute to mental health issues in children [17]. Additionally, a study by Reiss [18] indicated that positive parenting practices, such as emotional warmth and consistent discipline, can buffer the negative effects of socioeconomic hardships on children's mental health [18]. Despite these valuable insights, there remains a gap in understanding how these dynamics play out, specifically in the Australian context. This study addresses this gap using the Longitudinal Study of Australian Children (LSAC), a dataset that distinctively captures the nuances of Australian society. LSAC offers a unique perspective by tracking the development of Australian children over time, allowing for in-depth analysis. Its longitudinal nature provides insights into how these factors interact and influence each other across different stages of a child’s life, which cross-sectional studies cannot offer.

Globally, mental health issues among children and adolescents are a significant concern. According to the World Health Organization (WHO), 10% of children and adolescents experience mental health issues, with 50% of these issues starting by the age of 14 years [19, 20]. In Australia, the situation is no different, and mental health problems among adolescents are on the rise. In 2020, an estimated 31.25% of adolescents aged 12–17 years experienced mental health problems, an increase from 26.75% in 2018 [21]. Shockingly, 7 out of 10 children present with mental health complaints to pediatricians, indicating the severity of the issue [22]. Furthermore, over one million children in Australia are growing up in households where at least one parent has a mental illness [23]. This number is alarming, as one in five Australian children is living with a parent who has a mental health disorder, increasing their risk of experiencing socioeconomic hardship [24–27], substance abuse [28, 29], family conflict [30], and child abuse [31, 32].

Given this overwhelming evidence, this study aims to bridge a critical gap in the literature by examining the intersection of SES, parenting styles, and parental relationships and their distributional impact on the mental health of Australian children and adolescents from intact biological parent families, using data from the LSAC. We hypothesize that lower SES correlates with less favourable parenting styles and strained parental relationships, contributing to the heightened mental health challenges faced by children and adolescents from these backgrounds. This study contributes to the literature in several ways. First, it provides a nuanced analysis of how parenting styles and parental relationships affect socioeconomic disparities in mental health among Australian children and adolescents from intact biological parent families. Second, it evaluates the extent to which parental characteristics contribute to the mental health disparities observed between high and low SES groups. Finally, it investigated the temporal dynamics between parental factors and income-related mental health disparities, offering insights into potential policy interventions. By addressing these critical aspects, our study not only advances the understanding of the interplay between family dynamics and socioeconomic factors in mental health outcomes, but also lays the groundwork for targeted interventions aimed at mitigating mental health disparities among children and adolescents across different socioeconomic strata.

## Methods

### Conceptual framework

**Fig. 1:**
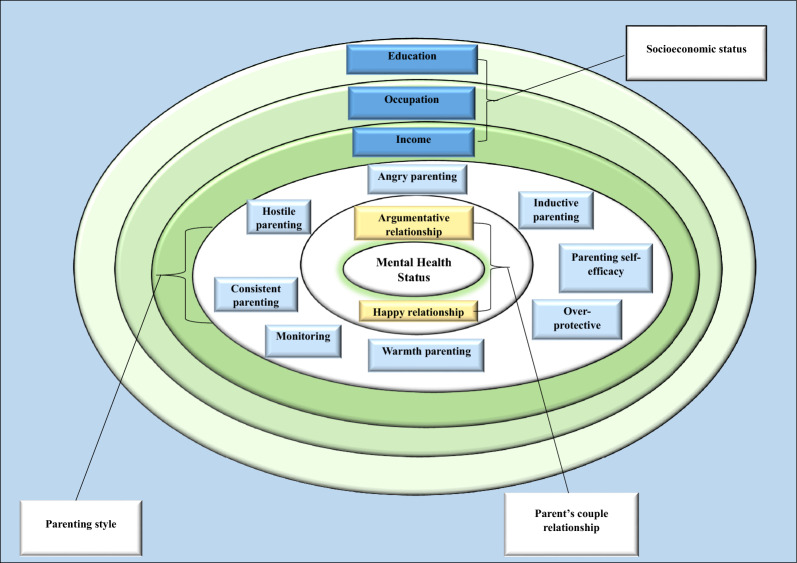
Conceptual framework socioeconomic status, parental style, parents couple relationship and mental health in children and adolescents

Within the conceptual framework of this study, our primary focus is on examining the distributional relationship between parenting style, parents' relationship, socioeconomic status (SES), and the mental health of children and adolescents. The framework combines eco-social theory and the social production of disease theory [58, 59]. The core of this paradigm is upon the recognition that individuals with lower socioeconomic status (SES) tend to experience higher levels of emotional distress as parents. This distress is typically marked by feelings of despair, worry, anger, and detachment. The distress may cause heightened disputes in the parental connection, which could lead to more severe, distant, or inconsistent parenting methods. The changes in parenting styles are essential in our framework since they have a direct correlation with the mental well-being of children and adolescents, as illustrated in Fig. 1.

### Study setting and study design

This study utilized data from waves one to seven of the LSAC dataset. LSAC is an ongoing, comprehensive, and multidisciplinary national representative survey that focuses on parenting, family relationships, education, employment, child health, and development. This survey used a multistage cluster sampling technique to collect the data. The data were collected from parents or caregivers (biological mother in 95% cases) of the children of participating households and from children themselves (from the age of 12 onward) through different methods (e.g., face-to-face interviews, self-reported questionnaires) with skilled interviewers. The detailed methodology for LSAC is available elsewhere [33]. In this study, we only used the K-cohort from Wave 1 to Wave 7 (i.e., aged 4–18 years) because of data availability. The baseline observation (n) of this cohort was 4953 and was followed up until wave 7 (n = 3014).

However, in the current study, children from single-parent families, adopted parents, stepparents, foster parents, aunts, and uncles were excluded from the analyses (Additional file 2). In this study, we only included children and adolescents from intact biological parent families. This decision stems from the understanding that children from step-blended or single-parent families often encounter unique challenges, including potential mental health burdens and socioeconomic inequalities that are not typically present in intact biological families. Across all seven waves, therefore, a total of 3014 children met these inclusion criteria for final analysis.

### Measure of mental health status

The mental health status of the children and adolescents was the outcome variable of this study. To measure mental health status, this study used the Strength and Difficulties Questionnaire (SDQ). The SDQ is a valid and reliable tool to measure the mental status of children and adolescents [34, 35] and has been used extensively to measure mental health status in children and adolescents [36–39]. The SDQ scores are based on five domains: hyperactivity, emotional problems, conduct problems, peer problems, and prosocial behaviours [40–42]. In this study, we used all domains except pro-social behaviors because of the unavailability of this variable in all waves of LSAC. Each of the four domains covered five items; for example, the hyperactivity scale (i.e., not being able to stay still, constantly fidgeting, being distracted, stopping to think before acting, good attention span), the conduct problems scale (i.e., temper, obeys requests, often fights, argumentative with an adult, spiteful to others), the emotional problems scale (i.e., complaints of headaches, seemed worried, unhappy, nervous, fearful), and the peer problems scale (i.e., solitary, liked by other children, bullied by children, gets on better with adults, has at least one good friend). These four domains were used to generate a total SDQ, and their response scales ranged from 0 to 40 [36]. Higher SDQ scores implied a negative mental health status (i.e., mental health difficulties/distress), whereas lower scores reflected a positive mental health status in children and adolescents.

### Measure of parental style

Parenting style is referred to as a collection of beliefs, values, and attitudes held by a parent regarding the health and development of children and teenagers [43–45]. A good parenting style (e.g., warm, consistent, supportive) has positive effects on a child's development; however, experiences of overprotection, rejection, and restriction by parents increase the risk of mental health issues in children and adolescents [46–48]. Thus, good parenting styles play a crucial role in the social-emotional development of children and adolescents [49, 50]. Based on established survey methods and theories, the LSAC integrated eight distinct parenting dimensions into their dataset: anger, inductiveness, consistency, over-protectiveness, parenting self-efficacy, monitoring, warmth, and hostility. However, our analysis omitted five of these dimensions: over-protectiveness, parenting self-efficacy, monitoring, warmth, and hostility, due to their limited availability in only one or two waves, which conflicts with our aim of conducting a comprehensive longitudinal study. Therefore, this study specifically focused on three dimensions: anger, consistency, and inductiveness in parenting. By examining the interplay between anger, warmth, and consistency, this approach aligns with Baumrind's 1991 framework in defining classic parenting styles [12]. Angry Parenting was measured using four questions, while Consistent Parenting and Inductive Parenting were measured using five and two questions, respectively. We calculated the frequency of exhibiting anger, consistency, or inductiveness towards children by computing the mean of the responses to the relevant questions on a 5-point Likert scale (1 = never, 5 = always) for each parenting style. Additional file 1 provides details of the questions used to measure parenting styles.

### Measure of parent’s relationship

A positive relationship between parents is defined as nurturing and investing in meaningful relationships for overall happiness and success which can be beneficial for the family psychological adjustment [51, 52]. However, negative relationships between parents increase conflict among family members and decrease emotional warmth, which has been significantly linked to developing emotional and behavioral problems in children and adolescents [17, 53–55]. Thus, a friendly home environment and a pleasant relationship between parents are important predictors of children’s health and development [56]. In this study, we used two indicators derived from LSAC data to measure the quality of parents’ couple relationships: the argumentative relationship scale and the degree of happiness in the relationship. The argumentative relationship scale is a four-question scale that assesses the level of conflict in a relationship, with higher values indicating a more argumentative relationship. The degree of happiness in a relationship is a single question that assesses the overall level of satisfaction with the relationship, with higher values indicating a happier relationship. For further details on the questions related to the parent-couple relationship, please refer to Additional file 1.

### Measure of income

Income was calculated as the sum of all members of a household's reported weekly income from all sources, which is referred to as disposable household income. We then used the Organization for Economic Co-operation and Development (OECD) equivalence scale to calculate equivalent household income [57]. Household income was used to measure SES and was constructed as the income component of the concentration index (CI). Equation (1) is used to calculate equivalent household income:

1 $${\text{Equivalent household income }} = \frac{{{\text{Household}}\,{\text{disposable}}\,{\text{income}}}}{{1 \times {\text{first}}\,{\text{adult}} + 0.5 \times {\text{additional}}\,{\text{adult}} + 0.3 \times {\text{additional}}\,{\text{child}}}}.$$

### Other variables

Mothers’ education and employment status within household income were used to control for other characteristics of the socioeconomic status of a household. Age, gender, and place of residence were also used as control variables in this analysis. The descriptive statistics of all the variables are provided in Table 1.

**Table 1:** Descriptive statistics of variables

Variables	Wave 1 (aged 4–5 years)	Wave 2 (aged 6–7 years)	Wave 3 (aged 8–9 years)	Wave 4 (aged 10–11 years)	Wave 5 (aged 12–13 years)	Wave 6 (aged 14–15 years)	Wave 7 (aged 16–17 years)	Pooled
*Mean*								
Dependent variable								
Mental health	9.34	7.9	7.46	7.94	7.45	7.11	7.24	7.78
*Independent variables*								
Parenting style								
Angry parenting	2.22	2.13	2.15	2.16	2.15	2.08	1.96	1.58
Praise behavior	3.79	3.61	3.72	3.68	3.61	3.63	3.67	3.67
Disapprove of behavior	2.59	2.29	2.41	2.44	2.39	2.31	2.14	2.36
Angry when punishing	2.62	2.31	2.44	2.51	2.41	2.24	2.06	2.37
Have problems managing	1.75	1.47	1.53	1.62	1.62	1.58	1.56	1.59
Consistent parenting	3.99	4.11	4.14	4.12	4.07	4.05	3.98	3.57
Make sure complete requests	4.31	4.12	4.24	4.21	4.11	4.05	3.88	4.13
Punish study child	3.88	3.79	4.12	3.97	3.89	3.81	3.52	3.85
Study children get away unpunished	2.25	1.95	2.11	2.04	2.02	1.98	1.92	2.03
Study children get out of unpunished	2.15	1.84	1.95	1.97	2.023	2.024	2.05	2.01
Study children ignore the punishment	1.95	1.63	1.55	1.63	1.64	1.61	1.63	1.65
Inductive parenting	4.17	4.14	4.05	4.07	3.95	3.77	3.21	3
Explain the corrections	4.42	4.26	4.18	4.22	4.09	3.91	3.42	4.06
Reason when misbehaves	4.26	4.12	4.21	4.23	4.13	3.93	3.31	4.02
Parents couple relationship								
Argumentative relationship	2.24	2.09	2.08	2.07	2.06	2.06	2.05	1.72
Disagreements re child-rearing	2.37	2.26	2.35	2.37	2.34	2.38	2.36	2.34
Stressful conversations	2.26	1.92	2.02	2.04	2.3	2.04	2.02	2.08
Arguments with partner	2.67	2.37	2.38	2.42	2.39	2.39	2.36	2.42
Hostility with partner	2.18	1.77	1.96	1.95	1.94	1.94	1.92	1.95
Happy couple relationship								
Degree of happiness with a partner	5.31	4.92	5.28	4.99	4.92	4.98	5.13	4.87
Household income								
Lowest income (500 AUD or less per week)	0.68	0.51	0.39	0.34	0.29	0.24	0.33	0.43
Lowest to medium (501–999 AUD per week)	0.25	0.39	0.45	0.46	0.45	0.46	0.35	0.36
Medium to highest (1000–1999 AUD per week)	0.05	0.08	0.11	0.17	0.23	0.26	0.26	0.17
Highest (more than 2000 AUD per week)	0.005	0.01	0.04	0.02	0.03	0.04	0.05	0.03
Mothers’ education status								
Postgraduation	0.06	0.07	0.07	0.08	0.09	0.09	0.09	0.08
Undergraduate	0.23	0.24	0.25	0.26	0.26	0.26	0.27	0.25
Certificate/Diploma	0.69	0.67	0.66	0.65	0.63	0.63	0.61	0.65
Year 12 or below	0.012	0.02	0.021	0.02	0.02	0.02	0.02	0.02
Mothers’ employment status								
Full-time employed	0.21	0.27	0.32	0.37	0.44	0.49	0.54	0.38
Part-time employed	0.36	0.41	0.42	0.39	0.36	0.33	0.31	0.36
Unemployed	0.42	0.33	0.26	0.23	0.19	0.17	0.15	0.25
Sociodemographic								
Age in years	0.26	0.39	0.35	0.42	0.50	0.45	0.45	0.35
Gender								
Male	0.50	0.50	0.50	0.50	0.50	0.50	0.50	0.50
Female	0.49	0.49	0.49	0.49	0.49	0.49	0.49	0.49
Areas of residence								
Accessible city areas	0.96	0.95	0.95	0.95	0.95	0.96	0.95	0.95
Not accessible regional areas	0.03	0.04	0.04	0.04	0.04	0.03	0.04	0.04

### Potential bias

Although cohort studies are generally less susceptible to bias than other observational methods, such as cross-sectional studies, it is crucial to acknowledge three specific types of potential bias in cohort studies: selection bias, informational bias, and confounder bias [58]. The LSAC data collection methods strictly adhere to the top international guidelines for longitudinal cohort studies, aiming to minimize biases related to geographical location and nonresponses [59, 60]. Although identifying every potential confounder bias is challenging, in this study, we used both crude and adjusted regression models to examine the influence of potential confounders (i.e., demographics) on the relationships between parenting style, parental couple relationship, SES, and mental health status.

### Statistical analysis

First, we employed descriptive statistics (frequency, mean, and standard deviation) to summarize the study variables. Second, using longitudinal data, we performed a wave-wise regression analysis to measure elasticities and examine the relationships between parenting style, parental couple relationships, and the mental health of children and adolescents. Subsequently, a concentration index (CI) was used to measure socioeconomic health inequalities in child and adolescent mental health. In addition, we applied the decomposition method to identify the factors contributing to the mental health status of children and adolescents (Additional file 2: Fig. S1). Furthermore, this study encountered some missing data that were addressed using a simple imputation method. All statistical analyses were conducted using R.

### Concentration index (CI)

The concentration index (CI) is a standard tool used to measure and quantify socioeconomic inequalities in health variables. It ranges from − 1 to 1 and indicates thea relationship between health variables and the standard of living. A negative (positive) value of concentration index (CI) exhibited that the health variable was more concentrated towards poor individuals (better off) and indicated a pro-poor (pro-rich) distribution. CI is calculated using the following equation [61, 62].

2 $$2\sigma_r^2 \left( {\frac{h_i }{{\mathop h\limits^- }}} \right) = \alpha + \beta r_i + \varepsilon_i$$

Here, $\sigma^2$ is the variance of the fractional rank, $h$ is the health variable interest of the study (i.e., mental health) population, $\overline{h}$ is the mean of health variable of interest, and $r_i \, = \frac{1}{N}$ is fractional rank of the study population rank by income or other indicators of socioeconomic status (i.e., $i = 1$ for poorest and $i = N$ richest).

### Decomposition analysis

To identify the contribution of each independent variable to socioeconomic inequalities in mental health status, this study used the Wafstaff, Doorslaer, and Watanabe approach to a decomposed the CI [63]. Wagstaff et al. [65] demonstrated that when health is considered as a linear function of various factors, demographics, parenting style, and socioeconomic status (SES), the concentration index (CI) can be expressed as a weighted sum of the socioeconomic inequalities observed in these factors. Therefore, the CI can be broken down based on the regression model. Equation (3) can be used to decompose the CI.

3 $$h_i = \alpha + \sum_i^k {\beta_k x_{ik} + \varepsilon_i }$$

where $\alpha$ is the intercept, $\beta$ is the coefficient, $X_k$ is a predictor, and $\varepsilon$ signifies the error terms. According to Wagstaff et al. [65], $CI$ of $h_i$ can be decomposed into the contribution of each predictor, which would explain its contribution to the distribution of mental health inequalities [63].

4 $$CI = \sum_k {\eta_k } CI_k + GC_u /\mu$$

where $\mu$ is the mean of the health variable, $\eta_k = \,\frac{\beta_k \chi_k }{\mu } = elasticity$, which measures the effect (positive and negative) of independent variables, $CI_k$ denotes the concentration index of each independent variable, and $GC_e$ denotes the error terms. Equation (4) gives the total contribution of socioeconomic inequalities explained by the model, where the error term shows the unexplained socioeconomic inequalities. The contribution percentage is calculated by $\left( {{{C_k } / {CI}}} \right) \times 100$. Furthermore, in this study, decomposition of the concentration index was calculated based on the following: first, we ran the multiple regression model (e.g., every wave from wave 1 to wave 7) by adjusting the parenting style, parent’s relationship, age of children and adolescents, gender, place of residence, mother’s education, mother’s employment, and household income. Secondly, we calculate the mean of the study variable, then the calculated mean of each variable was multiplied by the coefficient which was obtained from the regression model and got the elasticity. Thirdly, the concentration index was calculated using the library (rineq) packages in R, and the calculated concentration index was multiplied by the calculated elasticity to obtain the contribution of the variables. Moreover, for the pooled regression model, we applied the same steps as described above.

### Decomposition of concentration index change

This method analyses how the concentration of a particular variable changes over time. Therefore, in this study, we applied the Oaxaca and Blinder type decomposition approach to explain the differences in inequalities over a particular period [64, 65]. Although Wafstaff et al. [66] used this approach to investigate the factors that could change the health inequalities over the time. Applying the Oaxaca method to Eq. (4) yields the following equation:

5 $$\Delta CI = \sum_k {\eta_{kt} } \left( {C_{kt} - C_{kt - 1} } \right) + \sum_k {C_{kt - 1} } \left( {\eta_{kt} - \eta_{kt - 1} } \right) + \Delta \left( {GC_{e\,t} /\mu_t } \right)$$

where $t$ is the time period, $\Delta$ signifies the first differences, and the first and second terms indicate the extend of change in CI due to change in inequalities in the determinants of health and changes in their elasticity, respectively. The third term is residuals components. Moreover, the calculation of the decomposition of the concentration index change was based on the Oaxaca and Blinder approach: (i) estimate all elasticities and concentration indices of each using the prior methods, (ii) subtract all factors of current elasticities and concentration index from prior periods that gives the change in concentration index, (iii) each factor multiplies (wave wise) with changes in concentration indices and current period elasticities, and (iv) finally, by adding the current elasticity and concentration index, the total contribution to factor changes over the time period. The results are presented in an Additional file 1: Table S3.

## Results

### Descriptive results

Table 1 shows the descriptive statistics of the variables selected for this study from Wave 1 to wave 7. Parenting style and parent-couple relationships were the main variables of interest in this study. The average mental health status score (SDQ) of the Australian children and adolescents was 7.78. An average score of 7.78 suggests that, on average, the participants in this study have relatively low levels of mental health difficulties. Furthermore, the table shows that the mean of angry parenting, consistent parenting and inductive parenting were 2.22, 3.99, and 4.17 in wave 1 while 1.96, 3.98, and 3.21 in wave 7 respectively. Conversely, the mean values of argumentative relationships and happy couples’ relationships were 2.24 and 5.31 in wave 1, while this value reduced to 2.05, and 5.13 in wave 7, respectively. Higher average values for angry parenting, consistent parenting, and inductive parenting indicated more angry, consistent, and inductive parenting (Table 1). On the other hand, a higher average value of argumentative relationships implied more hostility with partners, while a higher average score of happy couple relationships indicated harmonious or happy relationships. The descriptive statistics are presented in Table 1.

### Regression results

Table 2 shows the regression results, estimated coefficients, and standard errors of the variables for Waves 1–7. Parenting styles (angry parenting, consistent, inductive) and argumentative relationships between parents were found to be statistically significant across the wave. The results suggest that on average, a one-unit increase in angry parenting increases the risk of mental health problems by more than three points (wave 1: *β*:3.017, wave 7: *β*:2.447) across the wave. Likewise, one-unit decrease in inductive parenting (wave 2: *β*:0.407; wave 7: *β*: 0.628) and a one-unit increase in parental argumentative relationships (wave 1; *β*:0.481; wave 7; *β*:0.371) also increased the risk of mental health difficulties across the wave. On the other hand, a one-unit increase in consistent parenting (wave 1: *β*: − 1.488; wave 7: *β*: − 1.184) and happy couple relationships (wave 2; *β*: − 0.14, wave 7; *β*: − 0.137) decreased mental health difficulties in Australian children and adolescents. This implies that consistent parenting and happy couple relationships between parents allow children and adolescents to feel a sense of safety and happiness. This positive environment is associated with better emotional well-being, lower stress levels, and improved mental health outcomes in children and adolescents.

**Table 2:** Regression results

Variables	Wave 1	Wave 2	Wave 3	Wave 4	Wave 5	Wave 6	Wave 7	Pooled
Independent variables	Co-eff (SE)	Co-eff (SE)	Co-eff (SE)	Co-eff (SE)	Co-eff (SE)	Co-eff (SE)	Co-eff (SE)	Co-eff (SE)
Parenting style								
Angry parenting	3.017 (0.147)***	2.637 (0.137)***	2.944 (0.143)***	3.331 (0.143)***	3.129 (0.135)***	3.144 (0.133)***	2.447 (0.153)***	2.9 (0.053)***
Consistent parenting	− 1.488 (0.135)***	− 1.268 (0.135)***	− 1.541 (0.140)***	− 1.140 (0.145)***	− 1.001 (0.137)***	− 1.179 (0.133)***	− 1.184 (0.135)***	− 1.286 (0.052)***
Inductive parenting	− 0.002 (0.133)	0.407 (0.122)***	0.363 (0.131)**	0.776 (0.124)***	0.719 (0.111)***	0.768 (0.099)***	0.628 (0.086)***	0.568 (0.039)***
Parents couple relationship								
Argumentative relationship	0.481 (0.184)**	0.689 (0.141)***	0.489 (0.171)**	0.790 (0.169)***	0.650 (0.161)***	0.490 (0.162)**	0.371 (0.166)*	0.613 (0.061)***
Happy couple relationship	0.027 (0.106)	− 0.14 (0.060)*	− 0.081 (0.098)	− 0.063 (0.082)	− 0.010 (0.079)	− 0.019 (0.080)	− 0.137 (0.080)*	− 0.045 (0.030)
Household income (ref: lowest income)								
Lowest to medium (= 1)	− 1.056 (0.209)***	− 0.586 (0.187)**	− 0.599 (0.186)**	− 0.803 (0.205)***	− 0.466 (0.205)*	− 0.031 (0.198)	− 0.092 (0.223)	− 0.531 (0.076)***
Medium to highest (= 1)	− 1.134 (0.445)*	− 1.556 (0.330)***	− 1.138 (0.290)***	− 0.849 (0.291)**	− 0.511 (0.254)*	− 0.438 (0.241)*	− 0.606 (0.261)*	− 0.866 (0.104)***
Highest (= 1)	− 0.105 (1.240)	− 0.808 (0.871)	− 1.712 (0.687)*	− 1.431 (0.713)*	− 0.967 (0.555)*	0.496 (0.492)	− 1.867 (0.485)***	− 1.135 (0.227)***
Mother’s education status (ref: postgraduate)								
Undergraduate (= 1)	0.047 (0.421)	0.073 (0.397)	− 0.449 (0.389)	− 0.327 (0.413)	0.055 (0.369)	− 0.130 (0.357)	− 0.346 (0.382)	− 0.182 (0.147)
Certificate/Diploma (= 1)	1.211 (0.399)**	0.952 (0.378)*	0.541 (0.370)	0.907 (0.392)*	1.137 (0.349)**	1.045 (0.338)**	0.527 (0.358)	0.858 (0.139)***
Year 12 or below (= 1)	0.753 (0.838)	0.954 (0.652)	1.223 (0.657)*	1.245 (0.757)	0.580 (0.774)	0.377 (0.723)	− 0.932 (0.720)	0.537 (0.274)*
Mother’s employment status (ref: full-time)								
Part-time Employed (= 1)	− 0.572 (0.236)*	− 0.303 (0.207)	− 0.41822 (0. 196)*	− 0.422 (0.210)*	− 0.258 (0.198)	− 0.158 (0.191)	− 0.060 (0.212)	− 0.260 (0.078)***
Unemployed (= 1)	0.26 (0.234)	− 0.027 (0.217)	0.25337 (0.222)	0.997 (0.240)***	0.860 (0.239)***	1.018 (0.228)***	1.350 (0.262)***	0.747 (0.088)***
Sociodemographic								
Age in years	− 0.236 (0.190)	0.265 (0.164)	− 0.054 (0.169)	− 0.034 (0.178)	− 0.110 (0.170)	− 0.113 (0.166)	0.164 (0.182)	− 0.038 (0.070)
Female (= 1) (ref: male)	− 1.070 (0.169)***	− 1.323 (0.161)***	− 1.279 (0.162)***	− 1.285 (0.177)***	− 1.125 (0.172)***	− 0.208 (0.166)	0.321 (0.183)	− 0.787 (0.065)***
Areas of residence (ref: accessible city areas)								
Not accessible regional areas (= 1)	0.428 (0.447)	0.348 (0.416)	0.166 (0.407)	0.81 (0.44)*	1.29 (0.431)**	1.082 (0.441)**	0.271 (0.483)	0.703 (0.167)***

(1) standard deviations (SD) are on parenthesis; (2) ‘*’, ‘**’ and ‘***” indicate statistical significance at 10%, 5% and 1% level. Lowest income = 500 AUD or less per week; lowest to medium (501–999 AUD per week), medium to highest (1000–1999 AUD), and highest income (more than 2000)

In the context of SES, Table 2 shows that children who belonged to medium-, high-, or highest-income households had lower mental health difficulties (with few exceptions) compared to children from the lowest income group. In the meantime, lower maternal education attainment (e.g., certificate/diploma;*β*:0.85,) shows an additional risk of mental health difficulties in children and adolescents. This signifies that children and adolescents from lower educated groups have a greater risk of mental health difficulties than their counterparts. Among other variables, this study found that female children had significantly fewer mental health problems (except Wave 7) than their male counterparts across the wave. Residential area was found to be a significant factor in mental health problems in Australia. Children in less accessible regions were more likely to have mental health problems than children living in cities or accessible areas across the wave.

### Decomposition of mental health inequality

Table 3 shows the factor decomposition of socioeconomic mental health inequalities among children and adolescents. In Table 3, the first row of each variable measures its elasticity (*η*). This implies that a change in the outcome variables is associated with a change in one unit of an independent variable. It had both negative and positive values. A negative (positive) value indicates a decrease (increase) in mental health difficulties relative to its predictors. The second row of each variable measures the concentration index (CI), which shows the direction of mental health inequalities. A negative (or positive) sign of the CI indicates that health (mental health status) is concentrated towards the poor (or rich) group and has led to a pro-poor (pro-rich) distribution. The third row represents the contribution of the factor to socioeconomic inequalities calculated by multiplying the elasticity within the concentration index.

**Table 3:** Decomposition (Wafstaff, Doorslaer, and Watanabe) results

Variables		Wave 1 (aged 4–5 years)	Wave 2 (aged 6–7 years	Wave 3 (aged 8–9 years	Wave 4 (aged 10–11 years	Wave 5 (aged 12–13 years	Wave 6 (aged 14–15 years	Wave 7 (aged 16–17 years	Pooled
Parenting style									
Angry parenting	$\eta$	0.717	0.711	0.848	0.906	0.903	0.922	0.662	0.589
	$CI$	− 0.0121	− 0.01	− 0.012	− 0.013	− 0.017	− 0.022	− 0.025	− 0.002
	$Con$	− 0.0087	− 0.009	− 0.010	− 0.012	− 0.016	− 0.021	− 0.017	− 0.0012
Consistent parenting	$\eta$	− 0.636	− 0.660	− 0.855	− 0.592	− 0.547	− 0.671	− 0.651	− 0.590
	$CI$	0.0148	0.01	0.009	0.015	0.013	0.008	0.013	0.002
	$Con$	− 0.0094	− 0.007	− 0.008	− 0.009	− 0.007	− 0.005	− 0.009	− 0.0010
Inductive parenting	$\eta$	− 0.001	0.214	0.197	0.398	0.381	0.407	0.278	0.219
	$CI$	− 0.0061	− 0.01	− 0.004	− 0.002	0.002	0.001	0.000	− 0.001
	$Con$	0.0000	− 0.002	− 0.001	− 0.001	0.001	0.001	0.000	− 0.0001
Parents couple relationship									
Argumentative relationship	$\eta$	0.115	0.182	0.136	0.206	0.180	0.142	0.105	0.134
	$CI$	− 0.0115	− 0.02	− 0.020	− 0.017	− 0.019	− 0.026	− 0.017	− 0.001
	$Con$	− 0.0013	− 0.003	− 0.003	− 0.003	− 0.003	− 0.004	− 0.002	− 0.0002
Happy couple relationship	$\eta$	0.015	− 0.086	− 0.057	− 0.039	− 0.007	− 0.013	− 0.097	− 0.028
	$CI$	0.0034	0.04	0.002	0.016	0.015	0.019	0.008	0.000
	$Con$	0.0001	− 0.003	0.000	− 0.001	0.000	0.000	− 0.001	0.0000
Household income									
Medium lowest (= 1)	$\eta$	− 0.029	− 0.027	− 0.034	− 0.041	− 0.024	− 0.002	− 0.004	− 0.025
	$CI$	0.6675	0.49	0.345	0.239	0.182	0.100	0.136	0.049
	$Con$	− 0.0194	− 0.013	− 0.012	− 0.010	− 0.004	0.000	− 0.001	− 0.0012
Medium highest (= 1)	$\eta$	− 0.006	− 0.017	− 0.020	− 0.018	− 0.016	− 0.016	− 0.022	− 0.019
	$CI$	0.9495	0.91	0.860	0.824	0.748	0.710	0.697	0.036
	$Con$	− 0.0059	− 0.016	− 0.017	− 0.015	− 0.012	− 0.011	− 0.015	− 0.0007
Highest (= 1)	$\eta$	0.000	− 0.001	− 0.004	− 0.004	− 0.004	0.003	− 0.013	− 0.004
	$CI$	0.9954	0.99	0.985	0.983	0.973	0.967	0.958	0.012
	$Con$	− 0.0001	− 0.001	− 0.004	− 0.004	− 0.004	0.003	− 0.013	0.0000
Mother’s education									
Undergraduate (= 1)	$\eta$	0.001	0.002	− 0.015	− 0.011	0.002	− 0.005	− 0.013	− 0.006
	$CI$	0.1905	0.27	0.261	0.269	0.219	0.220	0.211	0.012
	$Con$	0.0002	0.001	− 0.004	− 0.003	0.000	− 0.001	− 0.003	− 0.0001
Certificate/Diploma (= 1)	$\eta$	0.090	0.081	0.048	0.074	0.096	0.054	0.044	0.071
	$CI$	− 0.0773	− 0.11	− 0.106	− 0.117	− 0.109	− 0.111	− 0.107	− 0.007
	$Con$	− 0.0069	− 0.009	− 0.005	− 0.009	− 0.010	− 0.006	− 0.005	− 0.0005
Year 12 or below (= 1)	$\eta$	0.001	0.003	0.003	0.003	0.001	0.001	− 0.003	0.001
	$CI$	0.1447	− 0.02	0.003	0.058	− 0.051	− 0.008	− 0.016	0.052
	$Con$	0.0001	0.000	0.000	0.000	0.000	0.000	0.000	0.0001
Mother’s employment									
Part-time employed (= 1)	$\eta$	− 0.022	− 0.015	− 0.023	− 0.021	− 0.012	− 0.007	− 0.003	− 0.012
	$CI$	0.1049	0.08	0.042	0.018	− 0.003	0.011	− 0.022	0.001
	$Con$	− 0.0023	− 0.001	− 0.001	0.000	0.000	0.000	0.000	0.0000
Unemployed (= 1)	$\eta$	0.012	− 0.001	0.009	0.029	0.023	0.025	0.028	0.024
	$CI$	− 0.1928	− 0.25	− 0.297	− 0.294	− 0.344	− 0.283	− 0.364	− 0.028
	$Con$	− 0.0023	0.000	− 0.003	− 0.009	− 0.008	− 0.007	− 0.010	− 0.0007
Sociodemographic									
Age	$\eta$	− 0.007	0.013	− 0.003	− 0.002	− 0.007	− 0.007	0.010	− 0.002
	$CI$	− 0.0244	− 0.02	0.002	0.009	− 0.008	0.000	− 0.026	0.009
	$Con$	0.0002	0.000	0.000	0.000	0.000	0.000	0.000	0.0000
Gender									
Female (= 1)	$\eta$	− 0.057	− 0.082	− 0.085	− 0.080	− 0.074	− 0.014	0.022	− 0.050
	$CI$	− 0.0082	− 0.01	− 0.011	0.009	− 0.002	0.012	0.013	− 0.004
	$Con$	0.0005	0.001	0.001	− 0.001	0.000	0.000	0.000	0.0002
Areas of residence									
Not accessible regional areas (= 1)	$\eta$	0.002	0.002	0.001	0.004	0.007	0.006	0.002	0.004
	$CI$	0.0202	0.03	0.028	0.075	0.085	0.086	0.071	− 0.004
	$Con$	0.000	0.000	0.000	0.000	0.001	0.001	0.000	0.0000
CI of mental health (SDQ)	-	− 0.05	− 0.055	− 0.06	− 0.067	− 0.06	− 0.05	− 0.07	− 0.0045
Total estimated contribution	-	− 0.06	− 0.06	− 0.067	− 0.075	− 0.063	− 0.052	− 0.074	− 0.005

$\eta$ = elasticity; CI = concentration Index; con = contribution of the variable to socio-economic inequality

A negative value of the concentration index of angry parenting and argumentative parents’ relationships (Table 3) showed that poorer children and adolescents were more exposed to poor parenting styles (angry parenting) and poor parents’ relationships (argumentative relationships) than those with rich parental SES. The positive elasticities of angry parenting and argumentative relationships (Table 3) further indicate that angry parenting and argumentative parental relationships increase mental health problems and contribute to mental health inequality. Likewise, a positive CI for consistent parenting and happy relationships indicates that children from rich families experience consistent parenting and happy parental relationships, whereas a negative value of elasticity of consistent parenting and happy relationships (except for waves 3 and 5) implies that increasing consistent parenting and happy relationships would decrease mental health difficulties. Across the waves (excluding waves 1 and 2), the higher parental education attainment and household income groups had a positive concentration index and negative elasticity. This suggests that children and adolescents from higher socioeconomic backgrounds have better mental health status than their peers.

The second last, and last bottom row of Table 3 represent the CI of mental health status and the total estimated contribution of CI that describes the contribution to mental health inequalities, respectively. Table 3 (second last row) shows that socioeconomic inequalities of mental health ranged from − 0.05 to − 0.07 during the study period, which implies that poor mental health status was more concentrated in the pro-poor group and contributed to mental health inequalities in Australian children and adolescents.

**Fig. 2:**
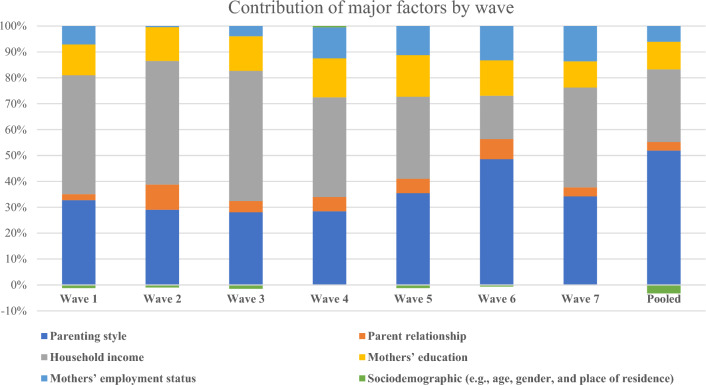
Contribution of major factors to socioeconomic inequalities in mental health by wave

This study revealed that the contribution of parenting style and parents’ relationships to mental health inequalities ranged from − 0.018 to − 0.025 and − 0.001 to − 0.003, respectively (see Additional file 1: Table S2). The contribution percentage was calculated by dividing each concentration index by the estimated concentration index and multiplying it by 100. Figure 2 shows that on average, parenting style, family income, maternal education, and maternal employment contributed to socioeconomic inequalities by 51.89%, 28.04%, 10.66%, and 9.28%, respectively. Thus, this study shows that parenting style is the most important factor contributing to socioeconomic health inequalities among Australian children and adolescents, followed by family income, maternal education, and mother’s employment status. This study also reveals that argumentative relationships between parents are a significant predictor of child mental health; however, their contribution to socioeconomic inequality is minimal (3.34%, see Fig. 2). The details are provided in the Additional file 2: Table S1.

### Factor trajectories of changes in mental health inequality

This study used the Oaxaca-Blinder approach to examine how mental health inequalities have changed over time (Additional file 1: Table S3). The findings of this study suggest that household income and parenting style are major factors of socioeconomic inequalities in the mental health of Australian children/adolescents. The highest household income increased mental health inequalities by 0.002 and 0.007 in waves 1–2 and 5–6, respectively. Subsequently, angry parenting increased inequalities by 0.005 in waves 6–7. The numbers "0.002" and "0.007" represent higher income quartile groups that have contributed to the increase in mental health inequality during two different periods (wave 1–2 and wave 5–6). Positive values suggest an increase in mental health inequalities. Subsequently, angry parenting also increased the inequalities by 0.005 in waves 6–7. Indicates the extent to which this variable contributed to the increase in mental health inequality due to the change in SES during waves 6–7. In summary, during the study period, the highest quartile income group consistently played a significant role in contributing to pro-rich mental health inequality, as measured using the Oaxaca-Blinder approach. Additionally, the "angry parenting" variable was a notable factor in increasing mental health inequality during a specific period. A list of the details is provided in Additional file 1: Table S3.

### Sensitivity analysis

The present study conducted sensitivity analyses using depressive feeling score as the dependent variable. The results were similar, and the basic interpretation remained the same. Thus, mental health inequality arising from parenting styles is a major driver of socioeconomic mental health inequality in the Australian context (see Additional file 1: Table S4).

## Discussion

The effect of socioeconomic inequalities on the mental health of children and adolescents is widely acknowledged, and reducing these inequalities has become a priority for policymakers in every country [65, 67]. However, the burden of mental health inequalities among young people appears to be increasing at an alarming rate; some even describe it as an epidemic [20]. Therefore, understanding the contributing factors and distribution patterns of mental health conditions is important for preventing and reducing mental health inequalities among children and adolescents [68]. This study aimed to identify and measure the factors correlated with socioeconomic inequalities and mental health among Australian children and adolescents from intact biological parent families, using data from waves 1–7 of the LSAC. The first objective of this study was to investigate the effect of parenting styles and the parents' couple relationships on the incidence of mental health issues in children and adolescents. The study found that a positive parental style and harmonious parental relationships have a significant and beneficial impact on the mental health status of children and adolescents. In addition, the findings of this study revealed a positive association between angry parenting and poor mental health outcomes in children and adolescents. It is suggested that a higher level of parental anger can lead to increased mental distress in parents, which in turn may cause them to become aggressive toward their children [17]. This aggression can have a detrimental impact on children's psychological well-being and overall health [69]. Earlier studies have also demonstrated that parental anger is not only associated with externalized manifestations but also has a severe impact on internalized problems for children and adolescents [70, 71]. Therefore, children who experience maltreatment, such as physical or emotional abuse or neglect, are at a higher risk of developing mental health problems both in childhood and later in life [72]. Furthermore, this study also demonstrated a positive relationship between poor inductive parenting and poor mental health status in children and adolescents. These findings have been mentioned in the previous literature, which highlighted that using poor inductive discipline techniques can hinder the development of empathy and guilt in children. Impacted children may not acknowledge the pain and distress they cause to others, which can increase their negative future behavior.

In contrast, this study highlights a negative correlation between consistent parenting practices and mental health problems among children and adolescents. This suggests that consistently implementing effective parenting strategies, such as monitoring, praising, and rewarding, to address unwanted behaviors can reduce the risk of psychological problems in children and adolescents. It is important to note that consistency in applying these strategies is a crucial factor in promoting positive outcomes and reducing distress in children and adolescents. Similar findings were documented in other studies, where parental consistent responsiveness, support, and warmth towards children and adolescents significantly reduced the level of psychological distress and increased positive feelings and emotions among children and adolescents [73, 74]. This study also revealed that an argumentative relationship between parents increases the probability of mental health problems in children, and conversely that parental harmony and happiness improve the mental health of their children. Relationships between parents that involve conflict and uncontrolled emotions can impede the emotional and behavioral development of their children, leading to a higher risk of mental health problems. In contrast, a happy parental relationship serves as a buffer against stressors and helps to maintain and develop the health of the entire family.

In concordance with other studies [75, 76], the findings of this study also indicate that children and adolescents from families with higher parental income and education levels are less likely to experience poor mental health than their peers from less advantaged backgrounds. This difference could be attributed to the availability of better-quality services and resources that have a positive impact on the mental health of young people. Place of residence was also positively correlated with mental health outcomes. Recent studies have suggested that urban planning with an emphasis on outdoor play spaces can be particularly beneficial for promoting and maintaining good mental health in young people [76, 77].

The second objective of this study was to examine socioeconomic mental health inequalities among children and adolescents by exploring the influence of parental characteristics, including parenting style, parental relationships, and parental SES. The findings of the study revealed that parenting style contributes to socioeconomic mental health inequalities among Australian children and adolescents by 51.89%, while couple relationships contribute by 3.34%. In addition, the study found a negative concentration index of angry parenting and argumentative parental relationships, which indicates that children and adolescents from poorer families are more exposed to poor parenting styles (angry parenting) and poor parental relationships (argumentative relationships) than those from more affluent families. Consequently, the study revealed a negative elasticity of consistent parenting and happy couple relationships. This implies that children and adolescents from richer families are more exposed to consistent parenting and happy parental environments. This positive relationship reduces the mental health consequences and contributes to mental health inequalities. On the other hand, a positive concentration index for consistent parenting and happy relationships indicates that children from rich families are more exposed to consistent parenting and happy parental relationships, which will reduce the mental health consequences and contribute to mental health inequalities. In addition, the positive elasticity of angry parenting and argumentative relationships further indicates that angry parenting and argumentative parental relationships increase mental health problems and contribute to mental health inequality in children and adolescents. Likewise, family income, mother’s education, and employment showed a similar effect on the distribution of socioeconomic mental health inequalities in Australian children and adolescents. The findings of this study are in line with previous research, indicating that economically disadvantaged parents may face significant challenges in providing their children with an intellectually and emotionally supportive environment. These challenges can lead to negative emotions, poor parent–child interactions, and inadequate nurturing, placing children and adolescents from low SES backgrounds at a heightened risk for poor mental health outcomes [78–80]. Additionally, children and adolescents who grow up in environments marked by inter-parental conflict are more likely to experience behavioral, social, emotional, and mental health problems [81–84].

The third objective of this study was to examine the temporal impact of parental characteristics, such as parenting style and parent–child relationships, as well as income-related mental health disparities over time (Additional file 1: Table S3). The findings suggest that angry parenting, inductive parenting, argumentative relationships, and household income were found to periodically intensify over time, exacerbating inequalities and contributing to the worsening of mental health inequalities. Therefore, understanding the root causes of these fluctuations is crucial for designing effective interventions to mitigate the mental health disparities among Australian children and adolescents. Overall, this study highlights the importance of parental characteristics and relationships in combination with household income in shaping mental health disparities among Australian children and adolescents. Therefore, multifactorial programs such as equitable mental health coverage programs, strengthening family and community support, family education and counselling services, and policies such as evidence-based action-oriented approaches, enhancing the services, particularly for deprived or underserved populations, are needed to address socioeconomic inequalities in Australian children and adolescents.

Despite a comprehensive investigation of the relationship between parental style, parental relationships, and socioeconomic inequalities in the mental health of children and adolescents, this study had some limitations. One primary concern is the potential impact of social desirability bias on the metrics reported by parents regarding the various aspects of parenting. There is a risk that parents may overstate their level of involvement and competencies with their children, influenced by the fear of being perceived as inadequate parents. Additionally, this study faces a significant limitation in the form of a higher attrition rate, which has the potential to influence the overall findings. Another notable limitation is the presence of missing data points for the study variables across waves, addressed through imputation. This introduces the possibility that such imputation may have influenced the findings. Nonetheless, despite this drawback, this study provides valuable insights into the factors that contribute to mental health disparities among young people in Australia, highlighting the importance of addressing socioeconomic inequalities to promote healthy lifestyles among Australian children and adolescents.

## Conclusions

This study investigated how and to what extent parenting style, parental relationships, and parental SES contribute to socioeconomic inequalities in the mental health of Australian children and adolescents from intact biological parent families. The accumulative novel findings of this study have shown that parental style and parental relationships significantly affect the mental health of children and adolescents. In addition, parents with low SES are more likely to practice poor parenting styles and have poor couple relationships, which contributes to mental health problems in their children and generates mental health inequalities among the study population. Early interventions are necessary to control the burden of mental health problems and build a healthy and harmonious home environment policy that can contribute to long-term socioeconomic health, including family health benefits for vulnerable populations, and the prevention of mental health problems, including the future prevention of chronic mental health disorders. Moreover, the findings of this study indicate that evidence-based intervention policies are needed to combat mental health problems experienced by children and adolescents living in poor parenting, inter-parental conflict, and poor SES groups.

### Supplementary Information

**Additional file 1.** Appendix A to Appendix D.

**Additional file 2.** Appendix E.

**Additional file 3.** Appendix F.

## Data Availability

The data used is confidential. Interested parties must comply with certain restrictions and sign confidentiality agreements. To request access to the data, individuals should reach out to the Australian Department of Social Services via the provided link: https://dataverse.ada.edu.au/dataverse/lsac.

## Abbreviations

CI: Concentration index
LSAC: Longitudinal Study of Australian Children
OECD: Organization for Economic Co-operation and Development
SES: Socioeconomic Status
SDQ: Strength and Difficulties Questionnaire
