# Supplementary material for: Socioeconomic inequalities in child and adolescent mental health in Australia: the role of parenting style and parents’ relationships
Source: Child Adolesc Psychiatry Ment Health. 2024 Feb 21;18:28. doi: 10.1186/s13034-024-00719-x (PMC10882797; doi:10.1186/s13034-024-00719-x)
Supplement: Supplementary file 1 — Additional file 1. Appendix A to Appendix D. [file 13034_2024_719_MOESM1_ESM.docx]

**Appendix A**

**Table 1: Contributing factors by percentage by wave**

| **Variables** | **Wave 1** | **Wave 2** | **Wave 3** | **Wave 4** | **Wave 5** | **Wave 6** | **Wave 7** | **Pooled** |
| --- | --- | --- | --- | --- | --- | --- | --- | --- |
| **Parenting style** | | | | | | | | |
| Angry parenting | 15.71% | 14.446% | 15.05% | 15.45% | 24.74% | 39.80% | 22.47% | 27.44% |
| Consistent parenting | 17.05% | 11.66% | 11.95% | 11.71% | 11.66% | 9.86% | 11.89% | 21.61% |
| Inductive parenting | -0.01% | 2.905% | 1.10% | 1.31% | -0.94% | -1.07% | -0.16% | 2.84% |
| **Couple (parents) relationship** | | | | | | | | |
| Argumentative relationship | 2.41% | 4.635% | 4.17% | 4.65% | 5.36% | 7.27% | 2.43% | 3.31% |
| Happy couple (parents) relationship | -0.09% | 5.108% | 0.14% | 0.83% | 0.16% | 0.48% | 1.05% | 0.03% |
| **Household income** | | | | | | | | |
| lowest to medium | 35.13% | 21.13% | 17.81% | 13.19% | 6.84% | 0.32% | 0.83% | 16.25% |
| Medium to highest | 10.65% | 24.87% | 25.99% | 20.08% | 18.48% | 21.89% | 20.65 | 10.8% |
| Highest | 0.12% | 1.765% | 6.45% | 5.23% | 6.41% | -5.46% | 17.1% | 0.99% |
| **Mothers’ education** | | | | | | | | |
| Undergraduate | -0.4% | -0.93% | 5.82% | 3.8% | -0.67% | 2.04% | 3.74% | 1.31% |
| Certificate/Diploma | 12.57% | 13.92% | 7.61% | 11.53% | 16.63% | 11.62% | 6.42% | 10.78% |
| Year 12 or below | -0.25% | 0.087% | -0.01% | -0.22% | 0.09% | 0.01% | -0.06% | -1.43% |
| **Mothers’ employment** | | | | | | | | |
| Part-time Employed | 4.22% | 1.83% | 1.48% | 0.5% | -0.06% | 0.15% | -0.08% | 0.2% |
| Unemployed | 4.12% | -0.44% | 3.93% | 11.44% | 12.54% | 13.74% | 13.89% | 9.08% |
| **Sociodemographic** | | | | | | | | |
| Age | -0.29% | 0.492% | 0.01% | 0.02% | -0.09% | 0.00% | 0.36% | 0.34% |
| Female (Ref: Male) | -0.85% | -1.39% | -1.45% | 0.94 | -0.21% | 0.33% | -0.38 | -3.89% |
| ***Areas of residence (Ref: accessible city areas****)* |  |  |  |  |  |  |  |  |
| Not accessible regional areas | -0.07% | -0.088% | -0.04% | -0.45% | -0.93 | -0.99% | -0.15% | 0.34% |
|  |  |  |  |  |  |  |  |  |

**Table 2: Contribution of major factors by concentration index**

| **Variables** | **Wave1** | **Wave2** | **Wave3** | **Wave4** | **Wave5** | **Wave6** | **Wave7** | **Pooled** |
| --- | --- | --- | --- | --- | --- | --- | --- | --- |
| Parental style | -0.018 | -0.018 | -0.019 | -0.021 | -0.022 | -0.025 | -0.025 | -0.002 |
| Couple relationship | -0.001 | -0.006 | -0.003 | -0.004 | -0.003 | -0.004 | -0.003 | -0.0002 |
| Household income | -0.025 | -0.031 | -0.033 | -0.029 | -0.02 | -0.009 | -0.028 | -0.001 |
| Mothers’ education | -0.006 | -0.008 | -0.009 | -0.01 | -0.01 | -0.007 | -0.007 | -0.0005 |
| Mothers’ employment | -0.004 | -0.001 | -0.004 | -0.009 | -0.008 | -0.007 | -0.01 | -0.0004 |

**Table 3: Oaxaca-Blinder decomposition**

| **Variables** |  | **Wave 1-2** | **Wave 2-3** | **Wave 3-4** | **Wave 4-5** | **Wave 5-6** | **Wave 6-7** |
| --- | --- | --- | --- | --- | --- | --- | --- |
| **Parenting style** | | | | | | | |
| Angry parenting | $\Delta C_{\eta}$ $\Delta\eta_{C}$ $T$ | -0.008  -0.002  -0.009 | 0.001  -0.002  -0.001 | -0.001  -0.0007  -0.002 | -0.004  0.0001  -0.004 | -0.005  0.000  -0.005 | -0.002  0.006  0.005 |
| Consistent parenting | $\Delta C_{\eta}$ $\Delta\eta_{C}$ $T$ | -0.006  -0.001  -0.007 | 0.002  -0.002  0.000 | -0.003  0.0039  0.001 | 0.001  0.0006  0.001 | 0.004  -0.001  0.003 | -0.004  0.000  -0.004 |
| Inductive parenting | $\Delta C_{\eta}$ $\Delta\eta_{C}$ $T$ | -0.002  0.000  -0.002 | 0.001  0.000  0.001 | 0.001  -0.0005  0.000 | 0.002  0.0000  0.002 | 0.000  0.000  0.000 | 0.000  0.000  0.000 |
| **Couple (parents) relationship** | | | | | | | |
| Argumentative parenting | $\Delta C_{\eta}$ $\Delta\eta_{C}$ $T$ | -0.003  -0.001  -0.004 | -0.001  0.001  0.000 | 0.001  -0.0012  0.000 | 0.000  0.0005  0.000 | -0.001  0.001  0.000 | 0.001  0.001  0.002 |
| Couple of degrees of happiness | $\Delta C_{\eta}$ $\Delta\eta_{C}$ $T$ | -0.003  -0.002  -0.005 | 0.002  0.000  0.002 | -0.001  0.0003  0.000 | 0.000  0.0005  0.000 | 0.000  0.000  0.000 | 0.001  -0.001  0.000 |
| **Household income** | | | | | | | |
| Medium lowest | $\Delta C_{\eta}$ $\Delta\eta_{C}$ $T$ | -0.013  -0.001  -0.014 | 0.005  -0.002  0.003 | 0.004  -0.0016  0.003 | 0.001  0.003  0.005 | 0.000  0.002  0.002 | 0.000  0.000  -0.001 |
| Medium highest | $\Delta C_{\eta}$ $\Delta\eta_{C}$ $T$ | -0.015  0.002  -0.014 | 0.001  -0.002  -0.001 | 0.001  0.0016  0.002 | 0.001  0.0020  0.003 | 0.001  0.000  0.000 | 0.000  -0.004  -0.004 |
| Highest | $\Delta C_{\eta}$ $\Delta\eta_{C}$ $T$ | -0.001  0.003  0.002 | 0.000  -0.003  -0.003 | 0.000  0.0004  0.000 | 0.000  -0.0002  0.000 | 0.000  0.007  0.007 | 0.000  -0.015  -0.015 |
| **Mothers’ education status** | | | | | | | |
| Undergraduate | $\Delta C_{\eta}$ $\Delta\eta_{C}$ $T$ | 0.001  0.002  0.003 | 0.000  -0.004  -0.004 | 0.000  0.0012  0.001 | 0.000  0.003  0.003 | 0.000  -0.001  -0.001 | 0.000  -0.002  -0.002 |
| Certificate/Diploma | $\Delta C_{\eta}$ $\Delta\eta_{C}$ $T$ | -0.008  -0.001  -0.009 | 0.000  0.004  0.004 | -0.001  -0.0030  -0.004 | 0.001  -0.002  -0.002 | 0.000  0.005  0.005 | 0.000  0.001  0.001 |
| Year 12 or below | $\Delta C_{\eta}$ $\Delta\eta_{C}$ $T$ | 0.000  0.000  0.000 | 0.000  0.000  0.000 | 0.000  0.0000  0.000 | 0.000  0.0001  0.000 | 0.000  0.000  0.000 | 0.000  0.000  0.000 |
| **Mothers’ employment status** | | | | | | | |
| Part-time employed | $\Delta C_{\eta}$ $\Delta\eta_{C}$ $T$ | -0.001  0.000  -0.001 | 0.001  0.000  0.000 | 0.001  0.0000  0.001 | 0.000  0.0000  0.000 | 0.000  0.000  0.000 | 0.000  0.000  0.000 |
| Unemployed | $\Delta C_{\eta}$ $\Delta\eta_{C}$ $T$ | 0.000  0.006  0.007 | 0.000  -0.003  -0.003 | 0.000  -0.0059  -0.006 | -0.001  0.002  0.001 | 0.002  -0.001  0.001 | -0.002  -0.001  -0.003 |
| **Sociodemographic variables** | | | | | | | |
| Age | $\Delta C_{\eta}$ $\Delta\eta_{C}$ $T$ | 0.000  0.000  -0.001 | 0.000  0.000  0.000 | 0.000  0.0000  0.000 | 0.000  0.0000  0.0002 | 0.000  0.000  0.000 | 0.000  0.000  -0.001 |
| Female | $\Delta C_{\eta}$ $\Delta\eta_{C}$ $T$ | 0.001  0.000  0.001 | 0.000  0.000  0.000 | -0.002  0.0000  -0.002 | 0.001  0.0000  0.001 | 0.000  0.001  0.001 | 0.000  0.000  0.000 |
| Not accessible regional areas | $\Delta C_{\eta}$ $\Delta\eta_{C}$ $T$ | 0.000  0.000  0.000 | 0.000  0.000  0.000 | 0.000  0.0003  0.000 | 0.000  0.0002  0.0003 | 0.000  0.000  0.000 | 0.000  0.000  0.000 |

**Note: $\Delta Cn$ signifies the change in health due to the changes in inequality, $\Delta\eta c$ implies the changes in health due to the changes in elasticity,**

**and $T$signifies the total changes that are explained by the determinants.**

**Table 4: Sensitivity analysis**

| **Variables** | Wave 5 | Wave 6 | Wave 7 | Pooled |
| --- | --- | --- | --- | --- |
| **Depressive feelings score (dependent variable)** | | | | |
| **Independent variables** | Co-eff (SE) | Co-eff (SE) | Co-eff (SE) | Co-eff (SE) |
| Angry parenting | 1.10  (0.15)  *** | 1.08  (0.06)  *** | 0.68  (0.22)  ** | 0.96  (0.11)  *** |
| Consistent parenting | -0.29  (0.15)  * | -0.40  (0.06)  *** | -1.24  (0.20)  ** | -0.36  (0.10)  *** |
| Inductive parenting | 0.19  (0.12)  * | 0.37  (0.04)  *** | 0.56  (0.13)  *** | 0.39  (0.07)  *** |
| Argumentative relationship | 0.37  (0.18)  ** | 0.19  (0.07)  ** | 0.53  (0.23)  ** | 0.31  (0.12)  * |
| Happy couple relationship | -0.19  (0.09)  * | 0.04  (0.03) | -0.14  (0.12) | -0.12  (0.06)  * |
| Lowest to medium (=1) | -0.26  (0.22) | 0.06  (0.08) | -0.22  (0.33) | -0.28  (0.16)  * |
| Medium to highest (=1) | -0.30  (0.28) | 0.05  (0.10) | -0.40  (0.38) | -0.34  (0.19)  * |
| Highest (=1) | 0.20  (0.61) | 0.38  (0.21)  * | -0.47  (0.71) | -0.34  (0.35) |
| Undergraduate (=1) | 0.06  (0.40) | -0.09  (0.15) | 0.03  (0.56) | 0.02  (0.25) |
| Certificate/Diploma (=1) | 0.86  (0.38)  * | 0.50  (0.14)  *** | 0.23  (0.52) | 0.48  (0.24)  * |
| Year 12 or below (=1) | 0.98  (0.85) | 0.18  (0.30) | -0.36  (1.05) | 0.24  (0.54) |
| Part-time Employed (=1) | -0.32  (0.22) | -0.04  (0.08) | -0.03  (0.31) | -0.10  (0.15) |
| Unemployed (=1) | 0.02  (0.26) | 0.21  (0.10)  * | 0.63  (0.18)  * | 0.24  (0.19) |
| Age in years | 0.12  (0.19) | 0.02  (0.07) | 0.25  (0.27) | 0.89  (0.04)  *** |
| Female (=1) (Ref: Male) | 0.71  (0.19)  *** | -0.75  (0.07)  *** | 2.47  (0.27)  *** | 1.73  (0.13)  *** |
| **Areas of residence (Ref: accessible city areas**) |  |  |  |  |
| Not accessible regional areas (=1) | 0.00  (0.47 | 0.35  (0.19)  * | -0.89  (0.71) | -0.42  (0.33) |

**Appendix: B**

**Parenting style**

**Angry parenting:** In LSAC, angry parenting was measured by following questions and these questions are available in all waves from Wave 1 to Wave 7

| **Question** | **Response** |
| --- | --- |
| Of all the times you talk to this child about his/her behaviour, how often is this praise? | 1 Never/Almost never; 2 Rarely; 3 Sometimes; 4 Often; 5 Always/Almost always |
| Of all the times you talk to this child about his/her behaviour, how often is this disapproval? | 1 Never/Almost never; 2 Rarely; 3 Sometimes; 4 Often; 5 Always/Almost always |
| How often are you angry when you punish this child? | 1 Never/Almost never; 2 Rarely; 3 Sometimes; 4 Often; 5 Always/Almost always |
| How often do you feel you are having problems managing this child in general? | 1 Never/Almost never; 2 Rarely; 3 Sometimes; 4 Often; 5 Always/Almost always |

**Consistent parenting:** In LSAC, consistent parenting was measured by following questions and these questions are available in all waves from Wave 1 to Wave 7

| **Questions** | **Response** |
| --- | --- |
| When you give this child an instruction or make a request to do something, how often do you make sure that he/she does it? | 1 Never/Almost never; 2 Rarely; 3 Sometimes; 4 Often; 5 Always/Almost always |
| If you tell this child he/she will get punished if he/she doesn't stop doing something, but he/she keeps doing it, how often will you punish him/her? | 1 Never/Almost never; 2 Rarely; 3 Sometimes; 4 Often; 5 Always/Almost always |
| How often does this child get away with things that you feel should have been punished? | 1 Never/Almost never; 2 Rarely; 3 Sometimes; 4 Often; 5 Always/Almost always |
| How often is this child able to get out of punishment when he/she really sets his/her mind to it? | 1 Never/Almost never; 2 Rarely; 3 Sometimes; 4 Often; 5 Always/Almost always |
| How often is this child able to get out of punishment when he/she really sets his/her mind to it? | 1 Never/Almost never; 2 Rarely; 3 Sometimes; 4 Often; 5 Always/Almost always |

**Inductive parenting:** In LSAC, inductive parenting was measured by following questions and these questions are available in all waves from Wave 1 to Wave 7

| **Questions** | **Response** |
| --- | --- |
| How often do you explain to this child why he/she is being corrected? | 1 Never/Almost never; 2 Rarely; 3 Sometimes; 4 Often; 5 Always/Almost always |
| How often do you talk it over and reason with this child when he/she misbehaves? | 1 Never/Almost never; 2 Rarely; 3 Sometimes; 4 Often; 5 Always/Almost always |

**Appendix: C**

**Parent’s couple relationship**

**Argumentative relationship scale:** In LSAC, an argumentative relationship was measured by following questions and these questions are available in all waves from Wave 1 to Wave 7

| **Questions** | **Response** |
| --- | --- |
| How often do you and your partner disagree about basic child-rearing issues? | 1 Never/Almost never; 2 Rarely; 3 Sometimes; 4 Often; 5 Always/Almost always |
| How often is your conversation awkward or stressful? | 1 Never/Almost never; 2 Rarely; 3 Sometimes; 4 Often; 5 Always/Almost always |
| How often do you argue with your partner? | 1 Never/Almost never; 2 Rarely; 3 Sometimes; 4 Often; 5 Always/Almost always |
| How often is there anger or hostility with your partner? | 1 Never/Almost never; 2 Rarely; 3 Sometimes; 4 Often; 5 Always/Almost always |

**Degree of happiness in relationship scale:** In LSAC, the degree of happiness in a relationship was measured by following questions and these questions are available in all waves from Wave 1 to Wave 7

| **Questions** | **Response** |
| --- | --- |
| Which best describes the degree of happiness, all things considered, in your relationship? | 1 Extremely unhappy; 2 Fairly unhappy; 3 A little unhappy; 4 Happy; 5 Very happy; 6 Extremely happy; 7 Perfectly happy |

**Appendix D:**

**STROBE Statement—Checklist of items that should be included in reports of *cohort studies.***

|  | Item No | Recommendation | Page No |
| --- | --- | --- | --- |
| **Title and abstract** | 1 | Provide in the abstract an informative and balanced summary of what was done and what was found | Page 2: L. No. 40 to 49 |
| Introduction | | | |
| Background/rationale | 2 | Explain the scientific background and rationale for the investigation being reported | Page 3 & 4: L. No 79 to 91 |
| Objectives | 3 | State specific objectives, including any prespecified hypotheses | Page 4: L. No. 102 to 106 |
| Methods | | | |
| Study design | 4 | Present key elements of study design early in the paper | Page 5: L. No. 129 to 133 |
| Setting | 5 | Describe the setting, locations, and relevant dates, including periods of recruitment, exposure, follow-up, and data collection | Page 5: L. No. 127 to 141 & See Appendix E |
| Participants | 6 | Give the eligibility criteria, and the sources and methods of selection of participants. Describe methods of follow-up | Page 5: L. No. 136 to 141 & Appendix E |
| Variables | 7 | Clearly define all outcomes, exposures, predictors, potential confounders, and effect modifiers. Give diagnostic criteria, if applicable | Page 5 to 8: L. No. 143 to 207 |
| Bias | 8 | Describe any efforts to address potential sources of bias | Page 7& 8: L. No. 204 to 207 |
| Study size | 9 | Explain how the study size was arrived at | See Appendix E |
| Quantitative variables | 10 | Explain how quantitative variables were handled in the analyses. If applicable, describe which groupings were chosen and why | Page 6 to 7:  L. No. 146 to 156,  L. No.162 to 173,  L. No. 181 to 186 |
| Statistical methods | 11 | Describe all statistical methods, including those used to control for confounding | Page 8 to 10:  L. No. 209 to 266 &  L. No. 204 to 207 |
| Results | | |  |
| Participants | 12 | Report numbers of individuals at each stage of study—eg numbers potentially eligible, examined for eligibility, confirmed eligible, included in the study, completing follow-up, and analysed | See Appendix E |
| Descriptive data | 13 | Give characteristics of study participants (eg demographic, clinical, social) information | See Table 1 |
| Outcome data | 14 | Report average numbers of outcome events | Page 10: L. No. 272 |
| Main results | 15 | Give unadjusted estimates and, if applicable, confounder-adjusted estimates and their precision (eg, 95% confidence interval). | Page 10 to 11: L. No 283 to 303 |
| Other analyses | 16 | Report other analyses done—eg analyses of subgroups and interactions, and sensitivity analyses | Page 11 to 13: L. No. 306 to 360 |
| **Discussion** | | | |
| Key results | 17 | Summarise key results with reference to study objectives | Page 13 to 15:  L. No. 370 to 376  L. No. 406 to 425  L. No. 430 to 432 |
| Limitations | 18 | Discuss the limitations of the study | Page 16: L. No. 441 to 448 |
| Generalisability | 19 | Discuss the generalisability (external validity) of the study results | Page 16: L. No. 452 to 462 |
| **Other information** |  |  |  |
| Funding | 20 | Give the source of funding and the role of the funders for the present study and, if applicable, for the original study on which the present article is based | NA |
