# Supplementary material for: Socioeconomic inequalities in child and adolescent mental health in Australia: the role of parenting style and parents’ relationships
Source: Child Adolesc Psychiatry Ment Health. 2024 Feb 21;18:28. doi: 10.1186/s13034-024-00719-x (PMC10882797; doi:10.1186/s13034-024-00719-x)
Supplement: Supplementary file 2 — Additional file 2. Appendix E. [file 13034_2024_719_MOESM2_ESM.docx]

**Appendix E**

**No. of non-mother Parents excluded***

**This Study Participants**

**Wave, Year**

**LSAC Participants**

**Ϯ** Loss to follow-up: n = 549

Attrition:18.8%

Attrition: 27.9%

Attrition: 7.6%

Attrition: 9.9%

Attrition: 12.8%

**Figure 1: Participant diagram**

*Non-mother parents are those who responded as parent 1 in LSAC and had a relationship with the study child as father, grandparent, adopted parent, stepparent, foster parent, aunt/uncle, sibling, or unrelated adults in the respective waves.

** All attritions are calculated from the first wave to the particular wave.

Ϯ Loss to follow-up has been calculated from the eligible sample of the first wave (baseline wave) to the particular wave.

Loss to follow-up: n = 1969

Loss to follow-up: n = 1484

Loss to follow-up: n = 1064

Loss to follow-up: n = 855

Loss to follow-up: n = 682

Mothers, n= 4953

Children, n = 4953

Mothers, n= 4434

Children, n = 4434

Mothers, n= 4301

Children, n = 4301

Mothers, n= 4128

Children, n = 4128

Mothers, n= 3919

Children, n = 3919

Mothers, n= 3499

Children, n = 3499

Mothers, n= 3014

Children, n = 3014

n= 38

n= 75

n= 30

n= 30

n= 30

n= 41

n= 38

Parents, n= 3089

Children, n = 3089

Parents, n= 3537

Children, n = 3537

Parents, n= 3957

Children, n = 3957

Parents, n= 4169

Children, n = 4169

Parents, n= 4331

Children, n = 4331

Parents, n= 4464

Children, n = 4464

Parents, n= 4983

Children, n = 4983

**Attrition: 5.52%

Wave 1, 2004

Wave 7, 2016

Wave 6, 2014

Wave 5, 2012

Wave 4, 2010

Wave 3, 2008

Wave 2, 2006
