# Supplementary material for: Socioeconomic inequalities in child and adolescent mental health in Australia: the role of parenting style and parents’ relationships
Source: Child Adolesc Psychiatry Ment Health. 2024 Feb 21;18:28. doi: 10.1186/s13034-024-00719-x (PMC10882797; doi:10.1186/s13034-024-00719-x)
Supplement: Supplementary file 3 — Additional file 3. Appendix F. [file 13034_2024_719_MOESM3_ESM.docx]

**Appendix: F**

Concentration index

**To measure socioeconomic health inequalities**

**Figure 1: Statistical analysis flow diagram**

Aggregate the data from Wave 1 to Wave 7

Wave by wave

Aggregate the data from Wave 1 to Wave 7

Wave by wave

Panel ordinary least square (POLS)

**To examine the relationships between parenting style, parental couple relationships, and the mental health of children and adolescents**

Regression analysis
